# Effect of altered proximal femoral geometry on predicting femoral stem anteversion in patients with developmental dysplasia of the hip

**DOI:** 10.1186/s13018-019-1491-4

**Published:** 2019-12-09

**Authors:** Chenhui Huang, Haitao Tan, Willem Alexander Kernkamp, Rongshan Cheng, Junjie Liang, Zhenan Zhu, Seung-Hoon Baek, Liao Wang, Tsung-Yuan Tsai

**Affiliations:** 1 Shanghai Key Laboratory of Orthopaedic Implants, Department of Orthopaedic Surgery, Shanghai Ninth People’s Hospital, Shanghai Jiao Tong University School of Medicine, School of Biomedical Engineering, Shanghai Jiao Tong University, Shanghai, 200030 China; 2 Guangxi Clinical Research Center for Digital Medicine and 3D Printing, Guigang City People’s Hospital, Guigang, 537100 China; 3 Engineering Research Center of Clinical Translational Digital Medicine, Ministry of Education of P.R. China, Shanghai, 200030 China; 4 Department of Orthopedic Surgery, School of Medicine, Kyungpook National University, Kyungpook National University Hospital, Daegu, 41944 South Korea

**Keywords:** Developmental dysplasia of the hip, Stem anteversion, Calcar femorale

## Abstract

**Background:**

The deformity of the proximal femur and acetabular in patients with developmental dysplasia of the hip (DDH) renders an intraoperative decision for ideal component placement challenging. We hypothesized that the altered morphology of calcar femorale (CF) in DDH patients changed the fixation mechanism of the cementless metaphyseal-filling stem and aimed to predict stem anteversion using proximal femoral anatomical parameters from preoperative CT.

**Methods:**

Preoperative and postoperative CT scans of 34 DDHs with a metaphyseal-filling stem in THA were retrospectively analyzed. Proximal femoral anatomical parameters, including the femoral anteversion (FA) and the CF angles at the low femoral neck (LFN) and the center of the lesser trochanter (CLT) levels (FA-LFN, FA-CLT, CF-LFN, and CF-CLT) were measured. The dysplastic hips were divided into the CF group (*n* = 21) and the non-CF group (*n* = 13) according to the presence of the CF-LFN. The association between the anatomical parameters and the postoperative stem anteversion was statistically analyzed, and the predicted stem anteversion was compared with postoperative stem anteversion.

**Results:**

In the CF group, the combination of the CF-LFN and FA-CLT exhibited a strong positive correlation (*R* = 0.870, *p* < 0.001) with the postoperative stem anteversion. In the non-CF group, only the FA-LFN had a strong positive correlation (*R* = 0.864, *p* < 0.001). Average prediction errors were 5.9° and 6.4° in the CF and non-CF groups.

**Conclusions:**

The presence of CF-LFN is related to the press-fit mechanism of the metaphyseal-filling stem, and the preoperative measurements from CT images can be employed as a tool to predict postoperative stem anteversion in DDH patients.

## Introduction

Dislocation has been reported as the main reason for revision after total hip arthroplasty (THA) [1] and is considered to be a more common postoperative complication in patients with developmental dysplasia of the hip (DDH). The reported dislocation rate after THA in DDH patients varies from 2.9% [2] to 9.5% [3]. Optimal placement of femoral stem is essential to minimize the risk of dislocation [4]. The concept of combined anteversion of the cup and stem has been generally accepted to improve stability and range of motion after THA [5, 6]. Dorr et al. [6] suggested to insert femoral stem first and then adjust acetabular cup anteversion to allow the combined anteversion within the “safe zone” of 25–50°. Patients with DDH are in a unique situation because of the increased anteversion in both proximal femur and acetabulum as a result of under-development [7, 8].

According to the concept of combined anteversion, in the case with increased stem anteversion, it was recommended for surgeons to decrease the cup version. Some surgeons, however, tend to place the cup in a more anteverted position matching with an increased version of the native acetabulum to maximize cup-host bone contact and thus, gain initial cup stability. Increased cup anteversion might lead to a potential risk for posterior impingement and anterior dislocation, whereas too decreased cup version exposes the hip to posterior dislocation and the cup to initial fixation failure [5].

Since the deformity of the proximal femur in DDH varies among individuals, and the severity of the disease, the resultant stem version also changes accordingly. This anatomical variation renders an intraoperative decision for ideal cup placement challenging. Thus, it is crucial to identify the preoperative anatomical parameters that associate with postoperative femoral stem anteversion. Such information to predict femoral stem anteversion with careful preoperative surgical planning together would be helpful for surgeons to decide the optimal cup position during THA to maximize joint stability and cup-host bone contact as well in DDH patients [9].

The calcar femorale (CF) plays an essential role in the initial stability and alignment of femoral stems [10] and has been reported to reduce torsional micromotion between the stem and host bone [11]. Several studies investigated the relationship between CF, femoral anteversion (FA) at various levels, and final stem anteversion using computed tomography (CT) scan in patients with primary osteoarthritis [9, 12–14]. However, CF, which provides essential structural support in the proximal femur, is shorter or even absent in some DDH patients [15] and previous studies, might be limited to apply for DDH patients with complex deformity. Thus, the effects of the altered morphology of CF in DDH patients on the postoperative stem anteversion in DDH patients remain unclear.

We hypothesized that the morphology of CF is associated with the press-fit mechanism of the cementless metaphyseal-filling stem and may determine the relationship between the endosteal surface and the postoperative stem anteversion in DDH patients. This study aims to predict anteversion of metaphyseal filling stem in patients with DDH using anatomical parameters of the proximal femur from preoperative CT and to evaluate the accuracy of the prediction using postoperative CT scan.

## Materials and methods

### Patient demographics

**Fig. 1:**
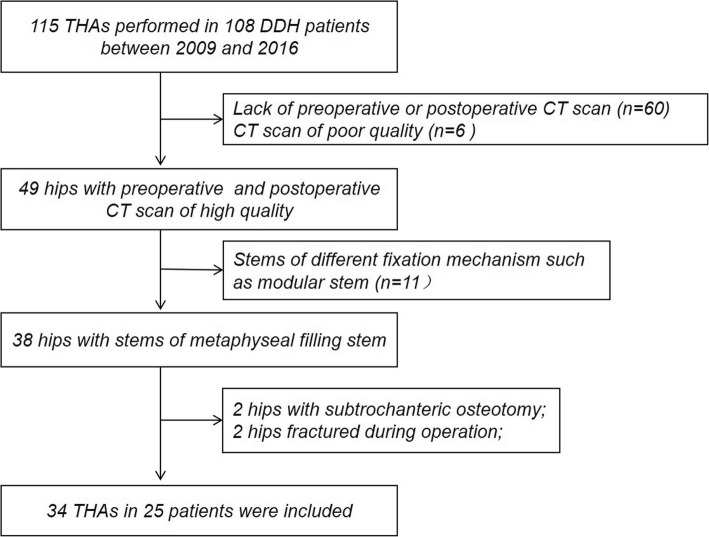
Flow chart diagram of patient selection

This retrospective study was approved from the Institutional Review Boards. Written informed consents were obtained from all participants. One hundred and eight DDH patients (115 hips) consecutively underwent primary cementless THA between December 2009 and April 2016 [16] (Fig. 1). The exclusion criteria were (1) patients who did not complete both preoperative and postoperative CT scans (60 hips), (2) those with CT scan of poor quality (6 hips), (3) those received different stem with different fixation mechanism (11 hips), and (4) those who underwent subtrochanteric osteotomy enabling the surgeon to modify stem anteversion (2 hips) or periprosthetic fracture affecting postoperative stem anteversion (2 hips). The remaining 34 THAs were included in this study. There were 5 males and 20 females with an average age of 63.8 ± 9.8 years (Table 1). The mean body mass index (BMI) was 24.0 ± 3.4 kg/m^2^. According to the classification of Crowe et al. [17], there were grade I in 22 hips, grade II–III in 8, and grade IV in 4. The mean follow-up duration was 5.6 ± 1.8 years. No patient demonstrated loosening, osteolysis, and dislocation at the final visit.

**Table 1:** The demographic data of the DDH patients

Parameters	All	CF group	Non-CF group	*p* value
Number, hips	34	21 (62%)	13 (38%)	NA
Age, years^#^	63.8 ± 9.8	64.9 ± 11.3	64.9 ± 6.3	0.985
Height, cm^#^	159.6 ± 5.7	159.5 ± 6.6	160.7 ± 4.4	0.602
Weight, kg^#^	61.2 ± 9.9	61.4 ± 8.7	60.9 ± 11.6	0.886
BMI, kg/m^2^^#^	24.0 ± 3.4	24.4 ± 3.2	23.0 ± 3.1	0.255
Crowe classification (I/II–III/IV)	22/8/4	18/2/1	4/6/3	0.004**

*DDH* developmental dysplasia of the hip, *CF* calcar femorale, *NA* not applicable, *BMI* body mass index

^#^Expressed as mean ± standard, ** and in boldface indicates statistically different

### Surgical technique and prosthesis

All THAs were performed using a posterolateral approach by a single senior surgeon (Z.Z.). Cementless metaphyseal-filling stem and hemispherical cup were implanted in all patients; Secur-Fit® stem and Trident® cup in 22 (Stryker, Mahwah, NJ, USA), and Summit® stem and Pinnacle® cup in 12 hips (Depuy, Warsaw, IN, USA). The surgical procedure followed a common modern technique called “combined anteversion.” The femoral neck osteotomy height follows the preoperative plan using the lesser trochanter as a landmark. Surgical broaches, gradually increasing in size, are used to prepare the femoral canal to maximize initial stability and osseous contact.

### CT scan and image processing

**Fig. 2:**
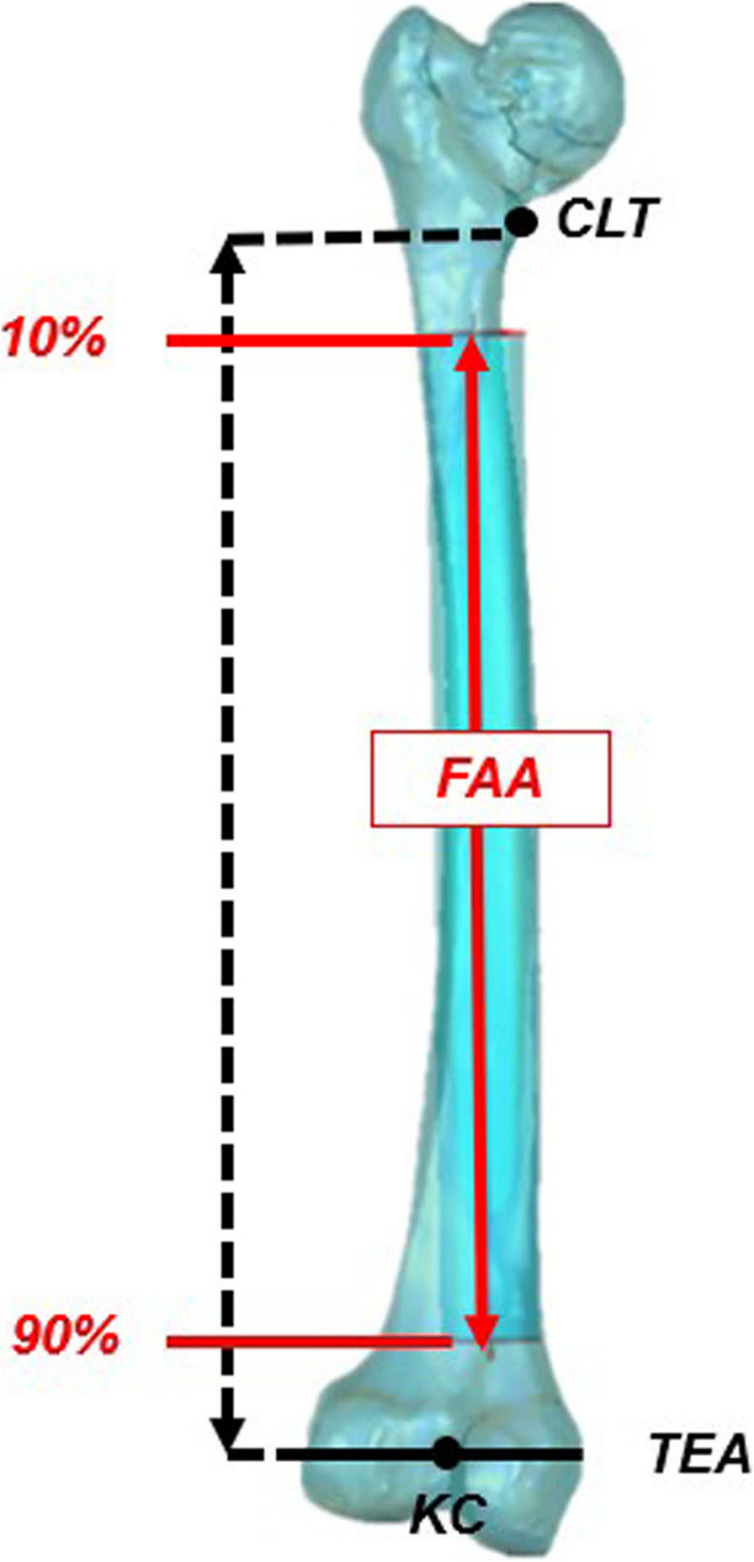
The definition of femoral anatomical axis (FAA). The FAA was defined as the center axis of a fitting cylinder from 10% to 90% of femoral length which was defined as the vertical distance between the center of the lesser trochanter (CLT) and knee center (KC). The KC was defined as the midpoint of the anatomical transepicondylar axis (TEA)

Postoperative CT scan of each patient was obtained postoperatively at 5.1 years on average (range, 1.9 to 8.8). The preoperative and postoperative CT images spanning from the fifth lumbar vertebra to distal femur were acquired using 128-slices CT scanners (Somatom Definition Flash®, Siemens Healthcare, Germany) with 1-mm slice thickness and in-plane resolution of 0.98 mm. High kilovolt was chosen to increase penetration and reduce starvation because of metal artifacts. The CT images were then imported into commercially available software (Amira®, Thermo Fisher Scientific, Waltham, MA, USA) [18] to construct 3D surface models of the preoperative and postoperative femur. To reduce the effects of arbitrary patient position during CT scanning, we chose the femoral anatomical axis (FAA) as the reference axis for the determination of the femoral anteversion (Fig. 2). The FAA was defined as the best-fit 3D line to the centroids of best fit circles to the outlines of the preoperative femoral shaft [19]. The postoperative femur was matched and aligned with the preoperative femur using surface-to-surface registration [20] to unify its reference. Both the preoperative and postoperative CT volume were resliced along the FAA with 1-mm slice thickness using Amira® software. Finally, the new resliced CT images were used to measure the anatomic parameters of the femur and the stem.

### Measurement of preoperative anatomic parameters and postoperative stem anteversion

**Fig. 3:**
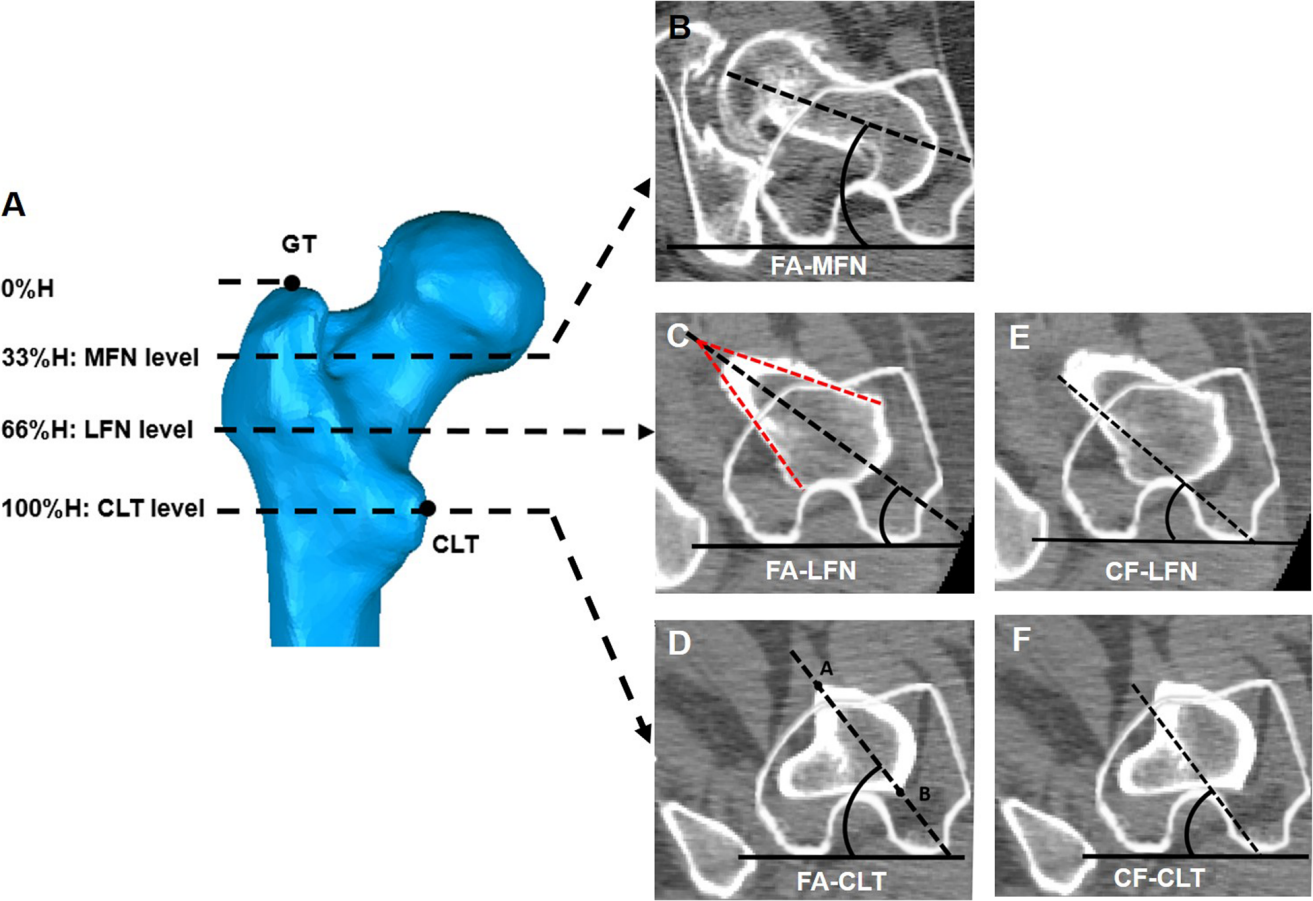
Measurement of proximal femoral anatomical parameters. **a** Three slice levels along the femoral shaft were taken. The proximal-distal distance between the proximal end of the greater trochanter (GT) and the center of the lesser trochanter (CLT) was defined as 100% proximal femoral height (H). The mid-femoral neck (MFN) level was located 33% distal to GT. The low femoral neck (LFN) level was located 66% distal to GT. The CLT level was taken at the level passing through CLT. The FA-MFN (**b**) and FA-LFN (**c**) were defined as the angle between the line (dotted line) bisecting the anterior and posterior cortex of the femur neck and PCA (solid line). The FA-CLT was considered equivalent to femoral canal major-axis torsion: the angle (major-axis angle) formed by the line (dotted line AB) connecting the longest transverse diameter of the canal and the PCA (solid line) (**d**). The CF-LFN (**e**) and CF-CLT (**f**) were defined as the angle between the PCA (solid line) and the line parallel to the CF (dotted line)

To account for the patient-to-patient size variation when measuring, we modified the selection of three slice levels purposed by Sugano et al. [7] and introduced a proximal femoral height parameter. The proximal-distal distance between the proximal end of the greater trochanter (GT) and the center of the lesser trochanter (CLT) was defined as the proximal femoral height (H). The three slice levels were determined along the FAA: the middle-femoral neck (MFN) level (33% of the H distal to the GT), the low femoral neck (LFN) level (66% of the H distal to GT), and the CLT level (passing through CLT) (Fig. 3a).

**Fig. 4:**
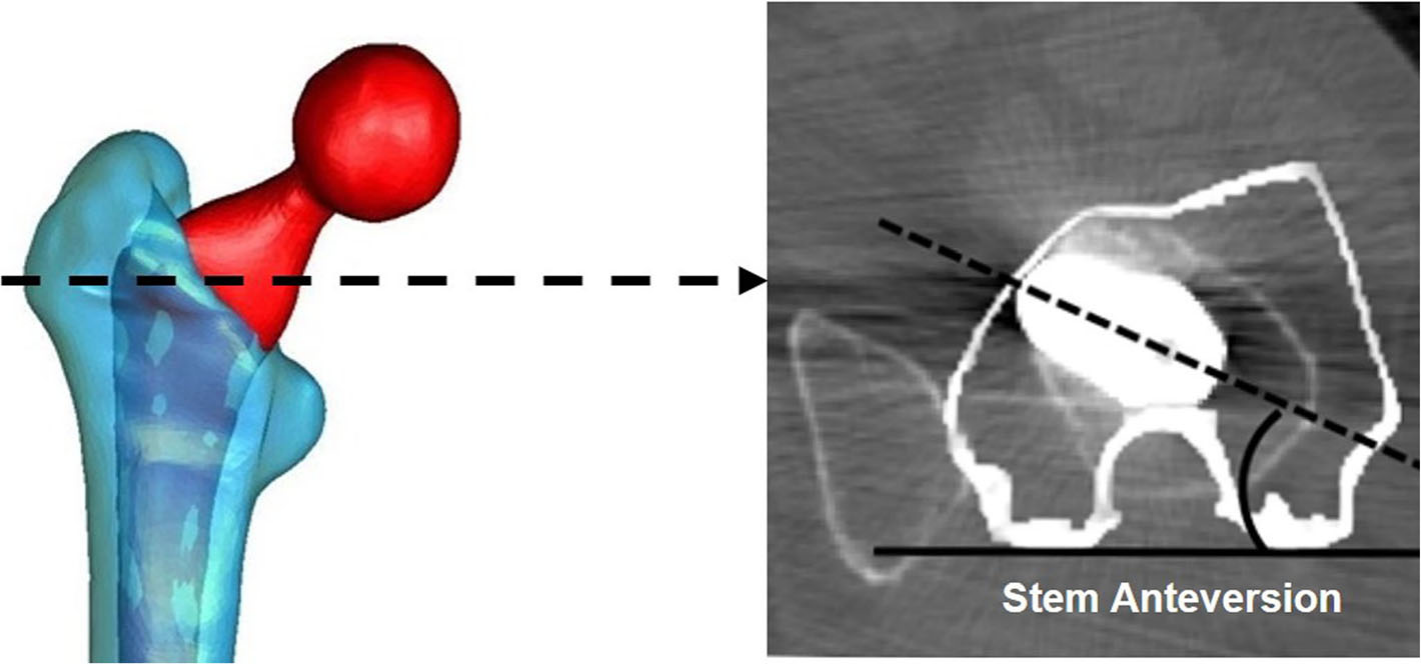
Measurement of postoperative stem anteversion. The postoperative stem anteversion was defined as the angle between the femoral stem neck axis (dotted line) and the PCA (solid line)

In the preoperative CT images, five proximal femoral anatomical parameters were measured: the FA at MFN, LFN, and CLT levels and CF angles at LFN and CLT levels (i.e., FA-MFN, FA-LFN, FA-CLT, CF-LFN, and CF-CLT) (Fig. 3b–f). The FA-MFN and FA-LFN were quantified as the angle between the line bisecting the cortex of the femur neck and the posterior condylar axis (PCA) at MFN and LFN levels [21] (Fig. 3b, c). The FA-CLT was considered equivalent to femoral canal major-axis torsion, that is the angle (major-axis angle) formed by the line connecting the longest transverse diameter of the canal and the PCA [7] (Fig. 3d). The CF-LFN and CF-CLT were defined as the CF angle between the PCA and the line parallel to the CF at LFN and CLT levels [15] (Fig. 3e, f). The postoperative stem anteversion was measured as the angle between the femoral stem neck axis and the PCA (Fig. 4).

Measurements were performed twice by two independent orthopedic surgeons (LW and JL). Pearson’s correlation analyses showed excellent intra-observer and inter-observer reliabilities (range, 0.847–0.969; 0.807–0.938).

### Patients grouping

The CF in some patients was thin and short [15], which was not clear to be identified on the CT slice at a relatively high level also, as the CF-LFN is closer to the contacting area between the femoral cortical bone and stem, which is more likely to affect the stem anteversion. We, therefore, divided the dysplastic hips into the CF and non-CF groups according to the presence of the CF-LFN.

### Statistical analysis

All continuous data were normally distributed, and hence, the data were expressed as means with standard deviations. The independent-sample *t* test was used for comparing continuous data between the CF and non-CF groups. The chi-squared test was used for categorical data. The Pearson product-moment correlation coefficient (*R*) was used to measure the strength of linear associations between the proximal femoral anatomical parameters and the postoperative stem anteversion. The multiple linear stepwise regression (forward selection) were used separately in the CF and non-CF groups for choosing the appropriate anatomic parameters in predicting postoperative stem anteversion. The statistical significance level (α) was set at 0.05.

## Results

Thirty-three out of 34 hips (97.1%) had CF-CLT, while 21 hips (61.8%) had CF-LFN, which divided the hips into the CF and non-CF groups (Table 1). No significant difference between the CF and non-CF groups was found in age, gender, height, weight, and BMI (*p* > 0.05) (Table 1). However, the CF and non-CF groups were significantly different in terms of severity of the disease, according to Crowe et al. [17] (*p* = 0.004). In the CF group, 1 out of 21 hips (4.76%) was classified into grade IV, while 3 out of 13 hips (23.1%) in the non-CF group were categorized into grade IV.

The FA-MFN was significantly higher in the non-CF group than the CF group (*p* = 0.001, 33.5° ± 13.1° and 18.5° ± 9.5°). No significant differences in the FA-LFN, FA-CLT, and the postoperative stem anteversion were demonstrated between the groups (*p* > 0.05) (Table 2).

**Table 2:** Comparison of postoperative anteversion and proximal femoral anatomical parameters between the groups. Statistically significant higher FA-MFN was noted in the non-CF group

Parameters	CF group^#^	Non-CF group^#^	*T*	*p* value
Stem Anteversion, °	26.7 ± 14.8	27.9 ± 15.0	0.229	0.820
FA-MFN, °	**18.5 ± 9.5**	**33.5 ± 13.1**	**3.870**	0.001**
FA-LFN, °	37.6 ± 12.6	39.2 ± 17.3	0.332	0.749
FA-CLT, °	52.0 ± 11.2	61.4 ± 17.1	1.945	0.061
CF-LFN, °	35.9 ± 15.7	N.A.	N.A.	N.A.
CF-CLT, °	51.5 ± 11.6	N.A.	N.A.	N.A.

*N.A.* not available

^#^Expressed as mean ± standard deviation, ** and in boldface indicates statistically different

In the CF group, strong positive correlations were demonstrated between the anatomic parameters and the postoperative stem anteversion (Table 3); the FA-MFN (*R* = 0.607, *p* = 0.004), the FA-LFN (*R* = 0.686, *p* = 0.001), the FA-CLT (*R* = 0.744, *p* < 0.001), the CF-LFN (*R* = 0.828, *p* = 0.001), and the CF-CLT (*R* = 0.811, *p* < 0.001). The slopes and intercepts in the regression formulas depicted the positive relationship between the preoperative anatomic parameters and the postoperative stem anteversion (Table 3). The multifactor linear regression analysis identified that the combination of the CF-LFN and FA-CLT has the strongest positive correlation with the postoperative stem anteversion (*R* = 0.870, *p* < 0.001) (Table 4). The coefficients of the multifactor linear regression equation were 0.559 and 0.464 for the CF-LFN and FA-CLT, respectively, suggesting a 1° increase in both the preoperative CF-LFN and FA-CLT could lead to about 1° greater postoperative femoral stem anteversion in DDH patients with CF.

**Table 3:** The Pearson’s correlation between the proximal femoral anatomical parameters and the postoperative stem anteversion at three levels in two groups. In the CF group, all anatomical parameters statistically correlated with the postoperative stem anteversion. In the non-CF group, only the FA-LFN had a strong positive correlation

		FA-MFN	FA-LFN	FA-CLT	CF-LFN	CF-CLT
The CF group	*R*	0.607	0.686	*0.744*	*0.828*	*0.811*
	*p* value	0.004**	0.001**	< 0.001**	0.001**	< 0.001**
Regression formula*	Slope	0.941	0.807	0.976	0.777	1.035
	*y*-intercept	9.311	− 3.578	− 24.034	− 1.155	− 26.621
The non-CF group	*R*	0.486	*0. 864*	0.494	N.A.	N.A.
	*p* value	0.092	< 0.001**	0.086	N.A.	N.A.
Regression formula*	Slope	0.556	0.749	0.433	N.A.	N.A.
	*y*-intercept	9.256	− 1.433	1.327	N.A.	N.A.

*N.A.* not available

*The regression formula of the proximal femoral anatomical parameters (*x*, *t*., FA, and CF) and the postoperative stem anteversion (*y*) at three levels (MFN, LFN, and CLT) in the CF and non-CF groups were presented as *y* = ax + *b*; *a*: slope, *b*: *y*-intercept

**Indicates statistically significant correlation (*p* < 0.05)

**Table 4:** Regression analysis of the combination of CF-LFN and FA-CLT and postoperative stem anteversion in the CF group (the regression formula was presented as *y* = ax_1_ + bx_2_ + *c*; *a*: FA-CLT coefficient, *b*: CF-LFN coefficient, *c*: *y*-intercept). A strong positive correlation was noted between the combination and the postoperative stem anteversion in the CF group

Variables in model	Coefficient	*p* value
FA-CLT (*a*)	0.464	0.034**
CF-LFN (*b*)	0.559	0.001**
Constant (*c*)	− 17.497	
Predictors	*R*	*R*^2^
CF-LFN	0.828	0.685
FA-CLT + CF-LFN	0.870	0.756

**Indicates a statistically significant correlation (*p* < 0.05)

In the non-CF group, only the FA-LFN level showed a strong positive correlation with the stem anteversion (*R* = 0.864, *p* < 0.001) (Table 3) and was also the only predictive factor for the postoperative stem anteversion in the multifactor linear regression analysis. A 1° increase in the FA-LFN could result in 0.749 degrees higher postoperative femoral stem anteversion in DDH patients without CF (Table 3).

The relationship between the preoperative proximal femoral parameters and postoperative stem anteversion in DDH patients was used as prediction equations and evaluated. The absolute prediction errors of the postoperative stem anteversion were 5.9° ± 4.1° and 6.4° ± 3.5° for the CF and non-CF groups, respectively.

## Discussion

The concept of combined anteversion of the cup and stem has been accepted to reduce dislocations in patients after THA. However, the deformity of the proximal femur and acetabular in DDH renders an intraoperative decision for ideal component placement challenging. Prediction of the postoperative femoral stem anteversion would be helpful for surgeons to decide the optimal cup position during THA to maximize joint stability and cup-host bone contact in DDH patients. The CF in the proximal femur forms the posterior wall of the reamed canal in the metaphysis and can reduce torsional micromotion of the stem after THA [11]. In our study, we divided the DDH patients into the CF and non-CF groups based on the presence of the CF-LFN. The combination of the CF-LFN and FA-CLT demonstrated a strong correlation with the postoperative stem anteversion in the CF group while the FA-LFN was strongly correlated to postoperative stem anteversion in the non-CF group. We also provided different prediction equations for both the CF and non-CF groups of DDH patients to estimate accurate postoperative stem anteversion. These suggested that the existence of CF-LFN might be associated with the press-fit mechanism of the metaphyseal-filling stem.

Previous studies assessed the association between several anatomic parameters at different femoral levels and the postoperative stem anteversion after THA in OA patients [9, 13, 14]. However, these studies were not in agreement with the parameters affecting the femoral stem version [9, 13, 14]. Hirata et al. [13] pointed out that the femoral canal version at CLT was the most closely approximated, but not significantly related to postoperative stem anteversion (37.9 ± 9.9° and 38.0 ± 11.2°, *p* = 0.885). Park et al. [9] found that FA-MFN and FA-LFN were correlated with postoperative stem anteversion (*r* = 0.681 and 0.781), but the FA-CLT was not considered in their study. Taniguchi et al. [14] found that the femoral anteversion at MFN, LFN, and CLT levels were all correlated with postoperative stem anteversion (*r* = 0.78, 0.76, and 0.66, respectively). In our study, all five parameters at the three slicing levels were positively correlated with the postoperative stem anteversion in the CF group, indicating that preoperative FA significantly affected the postoperative stem anteversion. However, although the FA-MFN was considerably higher in the non-CF group than that of the CF group, postoperative stem anteversion was not significantly different between the two groups. This may part from that most of the middle femoral neck structure were removed during THA procedure. Thus, our findings indicated that the real preoperative structures affecting the postoperative stem anteversion might be lower than the MFN level, i.e., the LFN and CLT levels.

A positive correlation between the CF and postoperative stem anteversion has been reported in OA patients [10, 11, 15]. In this study, we also found a strong positive correlation between postoperative stem anteversion and both CF-LFN and CF-CLT in the CF group, supporting that the CF angle influences the version of the effective femoral cavity. This finding corroborates with the hypothesis that CF maintains the stability of the cementless femoral stem [11]. Thus, it is recommendable to protect the CF structure as far as possible during THA procedure to avoid mechanical failure of the femoral stem. Furthermore, our findings indicate that the combination of the FA-CLT and CF-LFN might be used to better predict postoperative femoral stem version, hence to improve the accuracy of cementless THA preoperative planning in DDH patients with CF.

The deformity in patients with DDH in the proximal femur changes the force transmission within the femur, which affects the development and geometry of CF. Earlier studies have reported that DDH patients with Crowe II to IV have a shorter neck length with increased anteversion, lower height of the femoral head center, and absent or shorter CF than that in Crowe I [7, 15]. Our study using CT scan confirmed the findings in the previous studies that more hips in the non-CF group were graded as Crowe II to IV (Table 1) and had greater femoral anteversion at the MFN level when compared with those in the CF group (Table 2). Also, in the non-CF group, the CF structure was not clear at CLT level and was very thin at the LFN on the CT scan. In the absence of CF, the primary structure restrained to rotation by metaphyseal-filling stem might be the anterior and posterior cortex at femoral neck cutting level. This inference was supported by our data that only the FA-LFN showed a strong positive correlation with the postoperative stem anteversion in the non-CF group (Table 3). The geometric deformity of the CF may be one of the factors responsible for the different mechanism of fixation between the CF and non-CF groups.

Combined anteversion technique was proposed to compensate for abnormal component anteversion to reduce dislocation [6]. To achieve an appropriate combined anteversion for each case, surgeons adjust cup anteversion according to stem anteversion. Therefore, the stem anteversion is essential, and we believe that our study to predict femoral stem anteversion as a part of preoperative surgical planning would be helpful for surgeons to decide the balanced cup position intraoperatively to maximize joint stability and cup-host bone contact as well in DDH patients. Via accurate prediction of postoperative stem anteversion, surgeons can adjust the cup orientation preoperatively and intraoperatively according to the concept of combined anteversion [6]. Achieving optimal component placement with accurate preoperative surgical planning could benefit the long-term survivorship as well as a better range of motion and functional recovery. Of course, surgeons can adjust the stem anteversion during THA by using undersized stem during THA [22]. However, this is not only difficult in the proximal femur with narrow mediolateral dimension [14] or metaphyseal filling stems following proximal femoral geometry [16], but also not recommendable for optimal initial stem stability and longevity of the stem as well [23].

Several limitations can be noted in our study. First, 3D modeling for proximal femoral anatomic parameters in DDH patients cannot be generalized to clinical practice due to its high labor intensity. According to the findings in our study, three axial CT slices could be selected to evaluate the FA and CF angles, and predict the postoperative stem anteversion. Second, varus or valgus geometry of the hip might influence the CF, which was not taken into account. Other types of severe deformity might lead to different results. Third, only 13 hips were included in the non-CF group so that the power was relatively low, and a study with a larger sample size would be necessary for the future. However, post hoc analysis indicated 91.4% and 62.9% power for multifactor linear regression separately in the CF group and non-CF group. Lastly, our study was performed using two specific metaphyseal-filling stems with similar fixation mechanism. Thus, our findings cannot be generalized to others from different design or fixation mechanism.

In conclusion, preoperative measurements on CT images can be employed as a tool to predict postoperative metaphyseal-filling stem anteversion in patients with DDH. Whether CF presents at the LFN level may influence the selection of predictive equations for the postoperative stem anteversion. Combining the FA-CLT and CF-LFN could provide an accurate prediction of postoperative stem anteversion in DDH patients with CF. If the CF is absent, the FA-LFN is the most effective predictor for postoperative stem anteversion in DDH patients.

## Data Availability

The datasets used and analyzed during the current study are available from the corresponding author on reasonable request.

## Abbreviations

CF: Calcar femorale
CF-CLT: Calcar femorale angle at CLT level
CF-LFN: Calcar femorale angle at LFN level
CLT: Center of the lesser trochanter
DDH: Developmental dysplasia of the hip
FA: Femoral anteversion
FAA: Femoral anatomical axis
FA-CLT: Femoral anteversion at CLT level
FA-LFN: Femoral anteversion at LFN level
FA-MFN: Femoral anteversion at MFN level
GT: Greater trochanter
H: 100% proximal femoral height
KC: Knee center
LFN: Low femoral neck
MFN: Middle-femoral neck
TEA: Transepicondylar axis
